# Formaldehyde Exposure among Children: A Potential Building Block of Asthma

**DOI:** 10.1289/ehp.118-a131b

**Published:** 2010-03

**Authors:** Tanya Tillett

**Affiliations:** **Tanya Tillett**, MA, of Durham, NC, is a staff writer/ editor for *EHP*. She has been on the *EHP* staff since 2000 and has represented the journal at national and international conferences

Formaldehyde, a staple chemical in the manufacturing industry, is known to trigger acute adverse health effects such as skin, eye, nose, and throat irritation. Research on the human health effects of this compound has focused on a possible link between formaldehyde exposure and nasopharyngeal cancer. A new study reports the results of a meta-analysis of the literature examining a potential link between formaldehyde exposure and the prevalence of asthma in children **[*EHP*** **118:313–317; McGwin et al.]**.

Formaldehyde resins are used in the manufacture of furniture, clothing, carpeting, and pressed-wood products such as particle board and hardwood plywood paneling. The result is chronic human exposure to formaldehyde in many homes. The authors were inspired in part by concerns about formaldehyde exposure among displaced Gulf Coast residents living in temporary trailer housing after Hurricane Katrina.

The investigators analyzed data from 7 research articles concerning 5,930 total participants (364 with diagnosed asthma) that included actual formaldehyde measurements. Results of a fixed-effect model—meaning the model did not account for variation among the 7 studies—indicated the prevalence of asthma was 3% higher with each 10-μg/m^3^ unit increase in formaldehyde. A random-effects model—which did account for variation among studies—indicated a 17% increase in asthma with the same unit increase in exposure. When 1 unusually influential study was excluded from the meta-analysis, the authors estimated a 24% relative increase in asthma based on both fixed- and random-effects models. In addition, studies that measured formaldehyde exposure in schools suggested stronger associations than studies that examined home exposures.

The authors describe several limitations to the study, including the use of self-reported asthma information in some studies and the use of cross-sectional study design, which limits the ability of a study to tease out whether exposures preceded the development of asthma. Despite these limitations, the authors believe their results support a positive association between increased formaldehyde exposure and risk of childhood asthma. They note that future research regarding this health issue should focus on well-designed prospective epidemiologic studies.

## Figures and Tables

**Figure:**
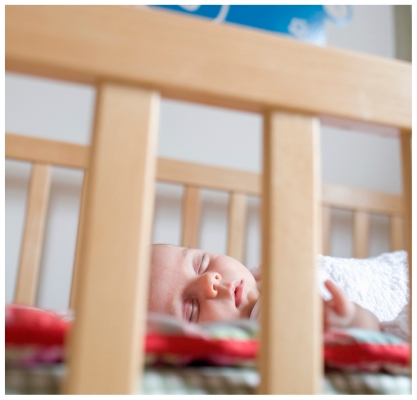
Formaldehyde is emitted by many manufactured wood products, which can contribute to high indoor exposures.

